# Association between Dietary Antioxidants and Atherosclerotic Cardiovascular Disease in South Korea: Insights from a Comprehensive Cross-Sectional Analysis

**DOI:** 10.3390/jcm13206068

**Published:** 2024-10-11

**Authors:** Jong-Ho Kim, Myeong Eun Lee, Sung-Mi Hwang, Jae-Jun Lee, Young-Suk Kwon

**Affiliations:** 1 Institute of New Frontier Research, College of Medicine, Hallym University, Chuncheon 24253, Republic of Korea; poik99@hallym.or.kr (J.-H.K.); melee@hallym.ac.kr (M.E.L.); 2 Big Data Center, Chuncheon Sacred Heart Hospital, College of Medicine, Hallym University, Chuncheon 24253, Republic of Korea; 3 Department of Anesthesiology and Pain Medicine, Chuncheon Sacred Heart Hospital, College of Medicine, Hallym University, Chuncheon 24253, Republic of Korea; h70sm@hallym.or.kr

**Keywords:** antioxidants, atherosclerosis, cardiovascular diseases, diet, national health and nutrition examination survey, South Korea

## Abstract

**Background/Objectives**: The multifactorial nature of atherosclerotic cardiovascular disease (ASCVD) implicates genetic, environmental, and dietary habits. Antioxidants found in foods have garnered attention for their potential role in mitigating ASCVD risk by combating oxidative stress. This study seeks to confirm the findings of previous research through a large-scale cross-sectional analysis performed in a unique population with Korea National Health and Nutrition Examination Survey data to explore the association between the composite dietary antioxidant index (CDAI) and ASCVD prevalence among middle- and old-aged individuals in South Korea. **Methods**: This study includes data from 2016 to 2021. The CDAI was calculated based on nutrition intake, including zinc, beta-carotene, vitamin A, vitamin C, vitamin E, and docosahexaenoic acid. This cross-sectional analysis explored the relationship between the CDAI and ASCVD after adjusting for relevant covariates. Logistic regression models were employed, and subgroup analyses by sex were conducted to discern sex-specific effects. **Results**: A total of 19,818 individuals were analyzed, with 7.0% of them diagnosed with ASCVD. CDAI distribution and antioxidant analyses revealed higher CDAI levels in non-ASCVD individuals. Standardized antioxidant values increased across CDAI quartiles. Initially, a significant association (odds ratio [95% confidence interval]: 0.96 [0.94–0.99]) was found between the CDAI and ASCVD, which was attenuated after adjusting for covariates (1.0 [0.98–1.02]). Subgroup analyses by sex showed nuanced associations, with the CDAI potentially reducing the risk of ASCVD in men (0.71 [0.53–0.94]) while increasing it in women (1.4 [1.01–1.95]). **Conclusions**: This study provides valuable insights into the association between dietary antioxidant intake and the risk of ASCVD, highlighting sex-specific differences.

## 1. Introduction

Cardiovascular diseases (CVDs) remain a major health challenge globally, contributing significantly to morbidity and mortality rates worldwide [1]. Among the various forms of CVD, atherosclerotic cardiovascular disease (ASCVD) stands out as a leading cause of myocardial infarction, stroke, and related complications [2]. It is a complex, and multifactorial condition influenced by genetic, environmental, and lifestyle factors [3,4,5]. As the prevalence of ASCVD increases, properly understanding the intricate interplay between these factors becomes imperative for the development of effective preventive strategies.

One of the key lifestyle factors that has garnered substantial attention in recent years is dietary habits [6]. New evidence suggests that the composition of one’s diet plays a pivotal role in ASCVD pathogenesis [7]. In particular, dietary antioxidants, which are found abundantly in fruits, vegetables, and various other food sources, have been investigated for their potential protective effects against oxidative stress, inflammation, and atherosclerosis [8,9,10,11]. Antioxidants are known to combat the damaging effects of free radicals, which are implicated in the development and progression of atherosclerotic lesions within arterial walls [12,13].

However, while numerous studies have explored the relationship between dietary antioxidants and ASCVD [8,9,10,12,13], the unique dietary patterns of different populations may result in varying associations between dietary antioxidants and ASCVD. Thus, there is a pressing need for region-specific investigations that shed light on the role of dietary antioxidants in the context of ASCVD.

Herein, we aimed to validate the findings of previous research by conducting a large-scale cross-sectional analysis using data from the Korea National Health and Nutrition Examination Survey (KNHANES), spanning the years from 2016 to 2021. We used the composite dietary antioxidant index (CDAI) to represent dietary antioxidants. This index is a measure of individual antioxidant profiles derived from dietary combinations, developed to assess and reflect the overall impact of dietary antioxidants on health. We assessed the association between the CDAI and the prevalence of ASCVD among middle-aged and older individuals in South Korea. By analyzing this extensive dataset, we aim to provide valuable insights into the potential impact of dietary antioxidant intake on the risk of ASCVD in the South Korean population.

## 2. Materials and Methods

### 2.1. Ethical Approval

This cross-sectional study was approved by the Clinical Research Ethics Committee of the Chuncheon Sacred Heart Hospital, Hallym University; because the risk to research subjects and the public was minimal, this study met the review exemption criteria set by Ordinance of the Ministry of Health and Welfare after review by the National Committee (IRB No. CHUNCHEON 2023-09-009).

### 2.2. Data Sources

The KNHANES [14] is a national survey that provides statistical data on the health status, health-related awareness and behaviors, and food and nutrition intake of the Korean population at the national and provincial levels. The survey is used to track the temporal trends of chronic diseases and related risk factors, and to provide evidence for health policy development, such as the establishment and evaluation of policy goals of the National Health Promotion Plan and the development of health promotion programs.

This survey has been conducted since 1998. The seventh KNHANES (2016–2018) included 4416 households in 192 survey areas nationwide, a total of 13,248 households with 24,269 individuals aged 1 or older. The eighth KNHANES (2019–2021) included 4800 households in 192 survey areas nationwide, a total of 14,400 households with 22,559 individuals aged at least one year. The survey items included a health questionnaire, health examination, and a nutrition survey. The seventh round included 274, 71, and 56 questions, respectively; while the eighth round included 203, 168, and 73 questions, respectively.

The survey results are weighted averages or weighted proportions of weighted samples. The weights are calculated to reflect the sample selection rate, response rate, and population composition of the target population in the survey year [14]. Our study included data from the seventh and eighth rounds of the KNHANES.

This study was a cross-sectional analysis of the Korea National Health and Nutrition Examination Survey (KNHANES) data spanning the years 2016 to 2021. It is crucial to clarify the nature of the abovementioned survey. KNHANES is a nationally representative survey conducted by the Korea Centers for Disease Control and Prevention. While the survey entails multiple rounds of data collection over three-year intervals, KNHANES is conducted annually. This annual schedule is intended to enhance the timeliness of national health and nutrition statistics, and the three-year intervals are determined by the survey’s design and logistical considerations.

KNHANES consists of health interviews, health examinations, and nutrition surveys. Health interviews and health examinations are conducted simultaneously in mobile screening vehicles, whereas the nutrition survey involves direct visits to the target households. It should be noted that health questionnaire items related to education, economic activity, morbidity, and medical use are collected through interviews, while items related to health behavior (such as smoking and drinking) are self-reported by the participants.

The sampling frame for the KNHANES is constructed using the latest available population and housing census data at the time of the sampling design. With this approach, it is certain to select a sample that is representative of the Korean population of people aged ≥ 1 year.

To clarify, KNHANES participants are not followed up longitudinally; rather, the survey captures a snapshot of their health and nutrition status at a given point in time. This cross-sectional design allows for data collection from a sample that is representative of the South Korean population at different time points, facilitating the monitoring of health, nutrition, and disease prevalence trends over time and providing valuable insights into the evolving health status of the population.

### 2.3. Study Participants

A total of 46,828 people participated in the seventh and eighth rounds of KNHANES. Middle and old age was defined as age ≥ 40 years based on survey items and previous reports [15,16].

### 2.4. Outcomes

Our primary outcome was ASCVD, which was defined as having at least one of the following diagnoses: coronary heart disease, angina, heart attack, or stroke, per the 2013 American College of Cardiology and American Heart Association guidelines for the treatment of hypercholesterolemia to reduce the risk of atherosclerotic cardiovascular in adults [17]. We determined ASCVD based on the diagnosis of myocardial infarction, angina, and stroke from a physician in the survey items.

### 2.5. Composite Dietary Antioxidant Index

The dietary intake survey assessed the participants’ dietary intake on the eve of the survey. KNHANES used the latest nutrient database available for each year to calculate nutrient intake. The CDAI is a composite score of various dietary antioxidants, including vitamins A, C, E, selenium, zinc, and carotenoids. The CDAI can reflect an individual’s profile and can indicate a person’s overall dietary antioxidant intake [18]. It is designed based on the demonstrable anti-inflammatory efficacy of dietary antioxidants according to their ability to attenuate inflammatory factors such as tumor necrosis factor-α and interleukin-1β [19]. We calculated the CDAI levels of all subjects using the modified version developed by Wright et al. [20]. The KNHANES includes zinc, beta-carotene, vitamin A, vitamin C, vitamin E, and docosahexaenoic acid as frequently consumed antioxidants. The index was calculated by normalizing each intake (subtracting the mean and dividing by the standard deviation) and then summing the antioxidants [8].

$$CDAI=\sum_{i=1}^{n=5}(Individual\;intake-Mean)/standard\;deviation$$

### 2.6. Other Variables

We used covariates to control for confounding factors and to improve the precision and interpretability of risk-adjusted models. Including covariates in the analysis can adjust for the effects of these confounding factors, and controlling for variables known to be associated with the outcome can reduce unexplained data variability, allowing for more accurate and precise estimation of the association between exposure and outcome [21,22]. Including risk factor-associated covariates can help to identify the unique contribution of the dietary antioxidant index and improve the model’s interpretability and applicability in real-world settings [23,24].

Covariate selection was determined based on our study’s objectives, the available data, and our prior knowledge of factors associated with ASCVD. The covariates included in our study were as follows: year of survey, age, sex, weight, waist circumference, body mass index, weight change over one year, household income quartiles, level of education, marital status, type of health insurance, drinking frequency over one year, amount of alcohol consumed in one sitting, taking at least one drink per month in the past year, lifetime cigarette smoking status, practicing aerobic physical activity, typical daily sitting time, number of days of vigorous physical activity (work and leisure), number of days of moderate-intensity physical activity (work and leisure), physical activity status (moving around), number of strength-training days per week, diagnosis of hypertension, dyslipidemia and diabetes by a licensed physician, glycated hemoglobin level, total cholesterol level, high-density lipoprotein level, triglyceride level, white blood cell count, and platelet count. Table A3 shows descriptions of these covariates.

### 2.7. Statistical Methods

Categorical variables were presented as counts and percentages, while continuous data were presented as the median and interquartile range (IQR). Absolute standardized differences were used to compare variables based on ASCVD status. Patients with incomplete or missing data were excluded from the analysis.

Our investigation involved exploring the relationship between the CDAI and ASCVD across the entire population and within male and female subgroups using scatter plots and box plots. Additionally, we examined the correlation between CDAI quartiles and individual antioxidants within male and female subgroups, using scatter plots and box plots.

Our dataset comprised a combined compilation from the seventh and eighth rounds of the KNHANES. To effectively amalgamate these rounds, we derived integrated weights by considering the health survey-examination-nutrition survey weights. The sampling technique employed in the KNHANES is a two-stage stratified cluster sampling method, emphasizing the necessity to account for this complex sampling structure when analyzing data. Consequently, our analysis results were generated while incorporating the complexities of strata, cluster, and weight factors.

For the assessment, we employed logistic regression models to compute the odds ratios (ORs) and 95% confidence intervals (CIs) for the CDAI concerning ASCVD. This analysis involved evaluating the CDAI both as a continuous variable and as an ordinal variable stratified into quartiles. For multivariable analyses, the following covariates were used: survey year, sex, age, household income quartiles, level of education, marital status, type of health insurance, diagnosis of hypertension by a licensed physician, diagnosis of dyslipidemia by a licensed physician, taking dyslipidemia medication, diagnosis of diabetes by a licensed physician, weight change over one year, drinking frequency over one year, amount of alcohol consumed in one sitting, taking at least one drink per month in the past year, average daily cigarette smoking, number of days of vigorous physical activity (work), number of days of moderate-intensity physical activity (work), physical activity status: moving around, number of days of vigorous physical activity (leisure), number of days of moderate-intensity physical activity (leisure), typical daily sitting time, number of strength-training days per week, practicing aerobic physical activity, weight, waist circumference, body mass index, glycated hemoglobin level, total cholesterol level, high-density lipoprotein level, triglyceride level, white blood cell count, and platelet count. Additionally, we used restricted cubic splines to confirm the association between the CDAI and ASCVD. Restricted cubic splines allow for more flexibility than simple linear regression.

Males typically exhibit a higher propensity for earlier onset of cardiovascular disease and have a heightened susceptibility to coronary heart disease [25]. Therefore, we conducted subgroup analyses to further investigate the relationship between ASCVD and the CDAI in males and females.

R (version 4.2.2; R Foundation for Statistical Computing, Vienna, Austria) was used for all statistics and graphics. Because we tested the CDAI in both continuous and nominal forms, involving multiple analyses, we set the alpha level at 0.025 (0.05/2) using the Bonferroni correction.

## 3. Results

### 3.1. Population Attributes

**Figure 1:**
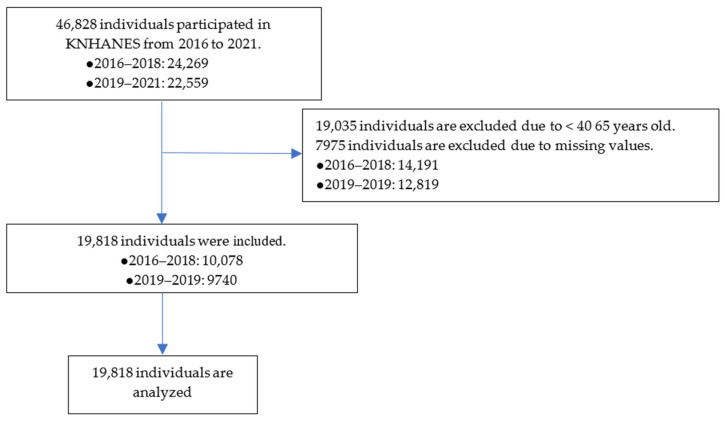
Flow chart of the participant inclusion process. KNHANES, Korea National Health and Nutrition Examination Survey.

The flow chart of the participant inclusion process is presented in Figure 1. The total population from 2016 to 2021 was 46,828, and individuals under the age of 40, or with missing values, were excluded, after which we ended up with 19,818 participants (10,078 from the seventh round and 9740 from the eighth round) included in the final analysis.

Table 1 summarizes the baseline features of the study participants, while Table A1 (Appendix A) provides an overview of the factors associated with medication use or lifestyle behavior. Of the 19,818 participants we included, 1382 (7.0%) were diagnosed with ASCVD. Significant absolute standardized differences of at least 0.25 were observed for age, sex, waist circumference, household income quartiles, level of education, hypertension, diabetes, dyslipidemia, ongoing dyslipidemia treatment, typical daily sitting time, number of days of vigorous physical activity (leisure), glycated hemoglobin level, total cholesterol level, and high-density lipoprotein level between participants with and without ASCVD.

### 3.2. The CDAI Distribution in Relation to ASCVD Presence/Absence and the Association of Individual Antioxidants with CDAI Quartiles

**Figure 2:**
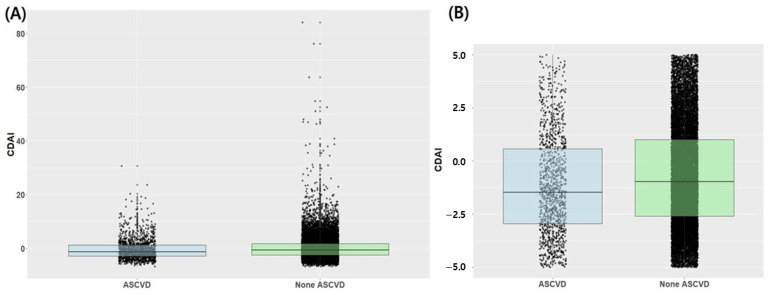
Box plot and scatter plot of CDAI according to the presence or absence of atherosclerotic cardiovascular disease. (**A**) Total population, (**B**) Expanded Focus: CDAI values ranging from −5 to 5; ASCVD, atherosclerotic cardiovascular disease; CDAI, composite dietary antioxidant index.

Figure 2 presents the distribution of the CDAI according to the presence or absence of ASCVD. The interquartile range of the CDAI in the ASCVD and non-ASCVD groups is −3.0 to 1.1 and −2.6 to 1.7, respectively. Notably, the 25th, 50th, and 75th percentile values of CDAI were higher in the non-ASCVD group compared with the ASCVD group. The 25th, 50th, and 75th percentile values of the CDAI are −2.60, −0.77, and 1.69, respectively.

**Figure 3:**
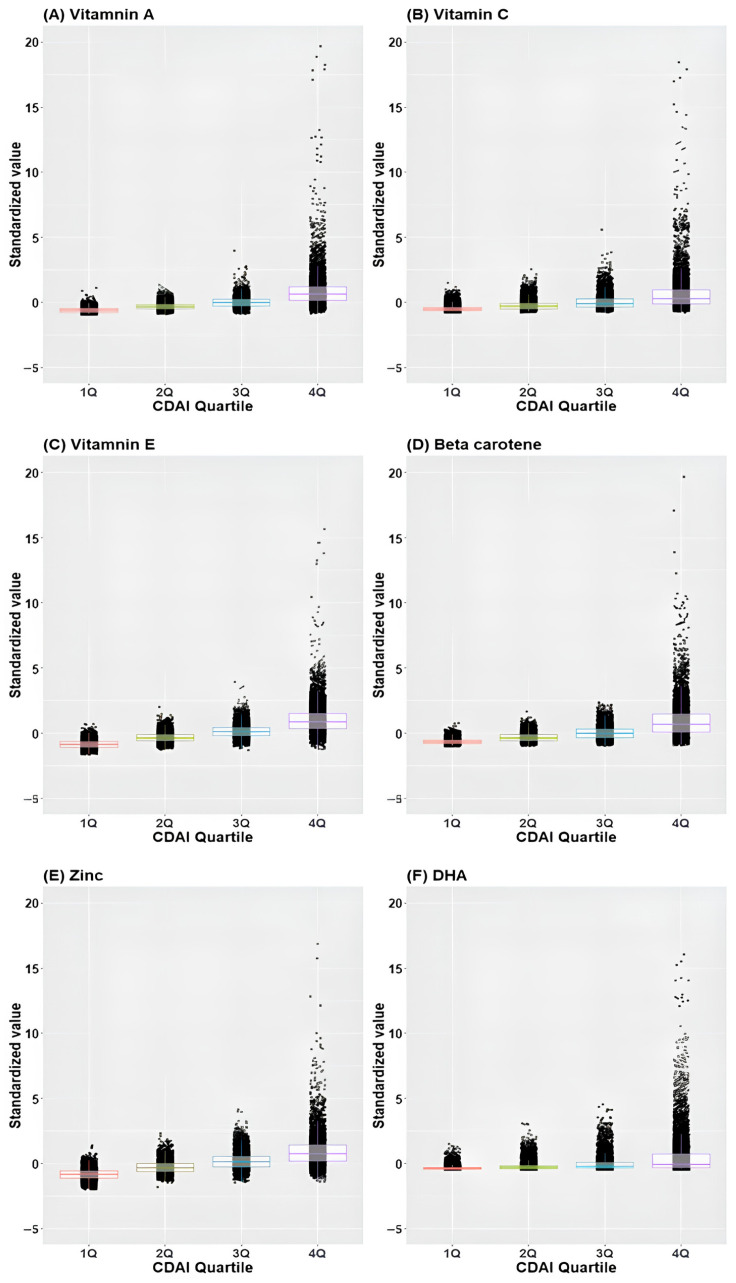
Box plot and scatter plot of standardized values of individual antioxidants across CDAI quartiles. (**A**) Vitamin A, (**B**) Vitamin C, (**C**) Vitamin E, (**D**) Beta-carotene, (**E**) Zinc, (**F**) Docosahexaenoic acid; CDAI, composite dietary antioxidant index; DHA, docosahexaenoic acid.

Figure 3 illustrates the distribution of standardized values ([individual intake − mean]/standard deviation) of individual antioxidants across CDAI quartiles. Remarkably, the standardized values demonstrated an incremental rise corresponding to higher quartiles across all antioxidants.

### 3.3. Odds Ratio of CDAI on ASCVD Prevalence

The significance of the association between the CDAI and ASCVD varied with covariate adjustment. Initially, without adjustments, a statistically significant association was observed between the CDAI and ASCVD (odds ratio [95% confidence interval]: 0.96 [0.94–0.99]). However, after adjusting for covariates, this association lost its significance (1.00 [0.98–1.02]). Regarding the CDAI quartiles, the second to fourth quartiles displayed a significantly reduced risk of ASCVD prevalence compared with the first quartile in the unadjusted analysis. However, after covariate adjustment, only the third quartile exhibited a significant reduction in the risk of ASCVD occurrence compared with the first quartile (0.77 [0.62–0.95]). For a more comprehensive overview, detailed results are presented in Table 2. Table A2 presents comprehensive details regarding the adjusted ORs and corresponding 95% CIs of covariates.

### 3.4. Subgroup Analysis: The CDAI Distribution in Relation to ASCVD Presence/Absence and the Association of Individual Antioxidants with CDAI Quartiles in Male and Female

**Figure 4:**
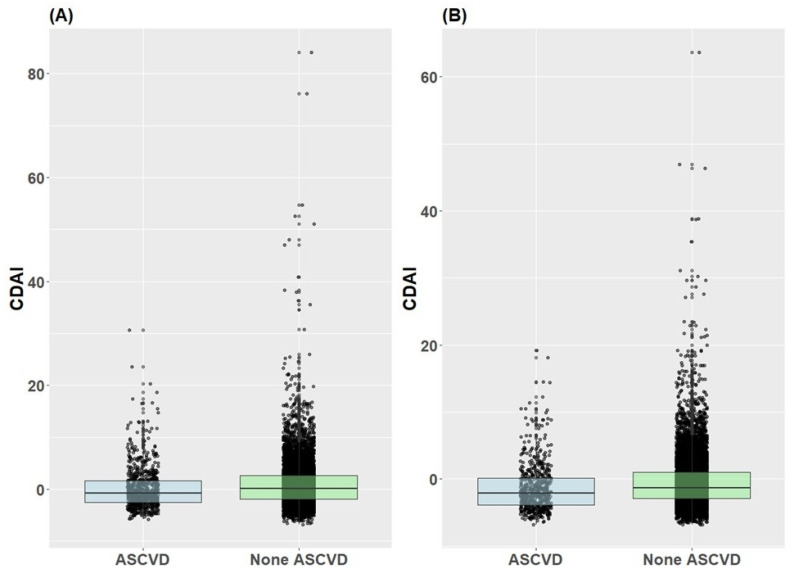
Distribution of CDAI by atherosclerotic cardiovascular disease presence/absence in male and female subjects. (**A**) Male, (**B**) Female.

Figure 4 displays the distribution of the CDAI according to the presence or absence of ASCVD in both male and female participants. For males, the interquartile ranges of the CDAI in the ASCVD and non-ASCVD groups were −2.5 to 1.7 and −1.8 to 2.6, respectively. The 25th, 50th, and 75th percentile values of the CDAI are −1.93, −0.03, and 2.53, respectively. For females, the interquartile ranges of the CDAI for the ASCVD and non-ASCVD groups were −3.8 to 0.1 and −2.9 to 1.0, respectively. The 25th, 50th, and 75th percentile values of the CDAI were −2.96, −1.32, and 0.93, respectively. Notably, the 25th, 50th, and 75th percentile values of the CDAI were higher in the non-ASCVD group than in the ASCVD group in both males and females.

**Figure 5:**
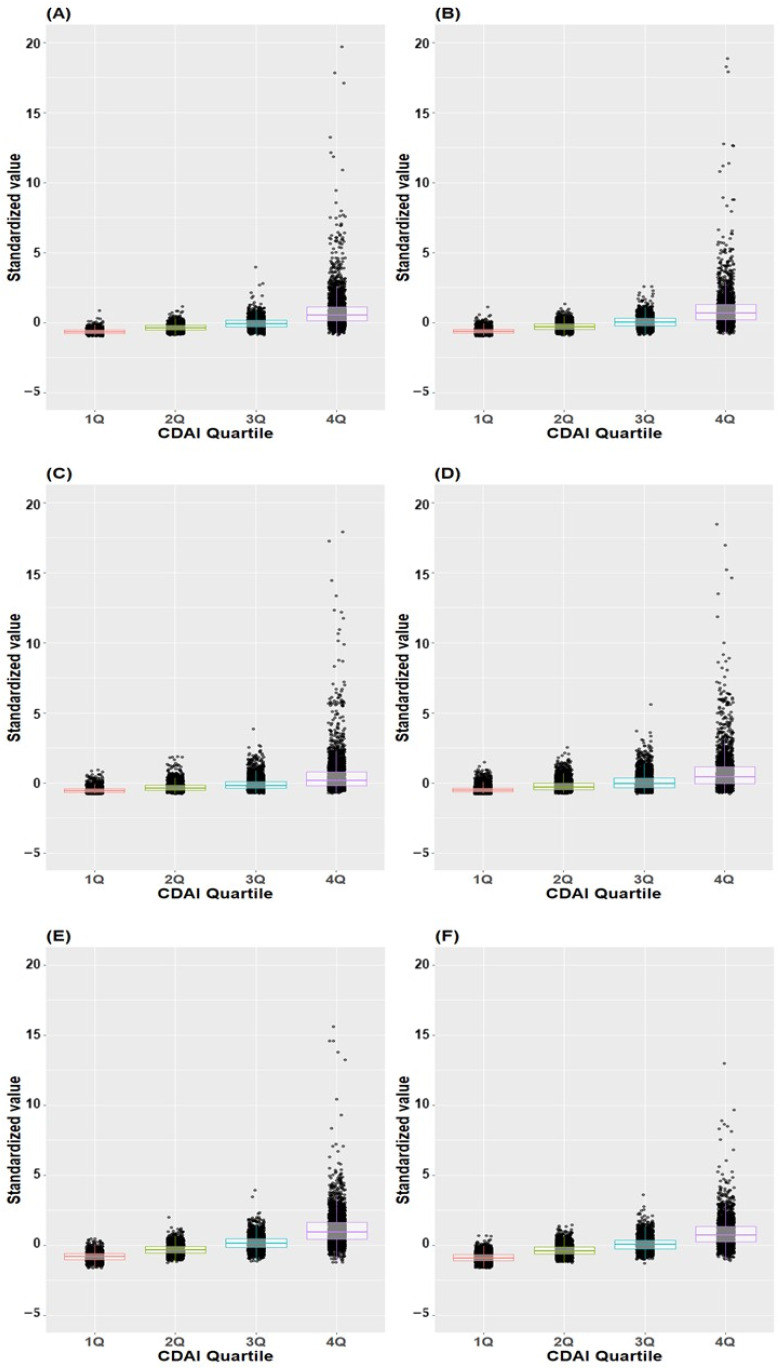

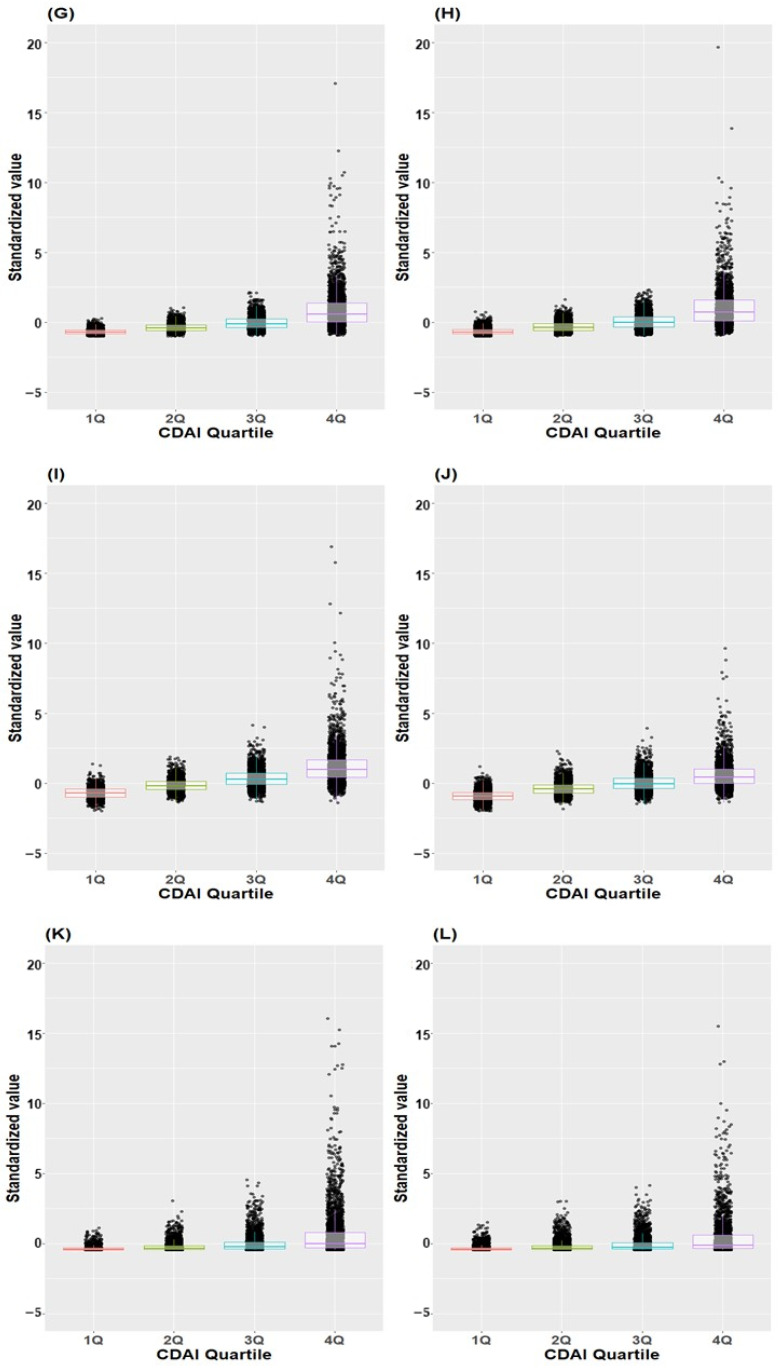
Box plot and scatter plot of standardized values of individual antioxidants across CDAI quartiles. (**A**) Vitamin A in males, (**B**) Vitamin A in females, (**C**) Vitamin C in males, (**D**) Vitamin C in female (**E**) Vitamin E in males, (**F**) Vitamin E in females, (**G**) Beta-carotene in males, (**H**) Beta-carotene in females, (**I**) Zinc in males, (**J**) Zinc in females, (**K**) Docosahexaenoic acid in males, (**L**) Docosahexaenoic acid in female; CDAI, composite dietary antioxidant index.

Figure 5 illustrates the distribution of standardized values for individual antioxidants across CDAI quartiles in both male and female subjects. The standardized values exhibited a progressive increase corresponding to higher quartiles across all antioxidants in both males and females.

### 3.5. Subgroup Analysis: Odds Ratio of CDAI on ASCVD Prevalence in Male and Female Populations

The association between the CDAI and ASCVD showed varying significance after adjusting for covariates in both males and females. Initially, a statistically significant association between CDAI and ASCVD (OR [95% CI]: 0.96 [0.91–0.97]) was found in males without adjustments. However, after adjusting for covariates, this association lost its statistical significance in both males and females. When analyzing CDAI quartiles, the second, third and fourth quartiles in unadjusted analyses among males displayed a significantly reduced risk of ASCVD occurrence compared with the first quartile. For females, the second and third quartiles exhibited a significantly reduced risk compared with the first quartile in unadjusted analyses.

After adjusting for covariates, the third quartile demonstrated a significant reduction in the risk of ASCVD occurrence compared with the first quartile in males (0.71 [0.53–0.94], *p* < 0.001). Although the *p*-value slightly exceeded the marginal threshold of alpha in the fourth quartile among males, it still indicated a reduction in the risk of ASCVD occurrence (*p* = 0.053). Conversely, for females, despite the *p*-value being slightly above the marginal threshold of alpha in the fourth quartile, it indicated an increased risk of ASCVD occurrence (*p* = 0.042). These results are outlined in Table 3.

### 3.6. Odds Ratio of CDAI on Stroke and Coronary Artery Diseases Prevalence

Table 4 shows the odds ratio of CDAI in stroke and coronary artery disease. It does not show consistent results in the association between an increased CDAI and disease prevalence when the CDAI is a continuous variable and when it is an ordinal categorical variable. In stroke, there is no significant relationship between CDAI and continuous variables. In coronary artery disease, there was no significant relationship when it was a continuous variable; however, when it was an ordinal categorical variable, the second and fourth quartiles showed lower incidence rates than the first quartile.

### 3.7. Association between CDAI and ASCVD Using Restricted Cubic Spline

**Figure 6:**
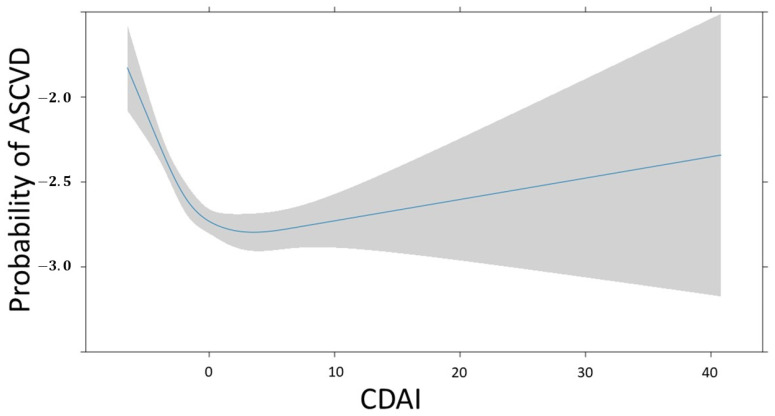
Restricted cubic spline fit of CDAI on ASCVD. The probability of ASCVD is expressed as log odds.

Figure 6 depicts the association between CDAI and ASCVD. The change in ASCVD prevalence follows a non-linear pattern as the CDAI increases. The probability is high when the CDAI is below 0, then it gradually decreases when it is around 0, and subsequently increases as it continues to rise. The confidence interval for the probability of ASCVD widens as the CDAI surpasses 0.

**Figure 7:**
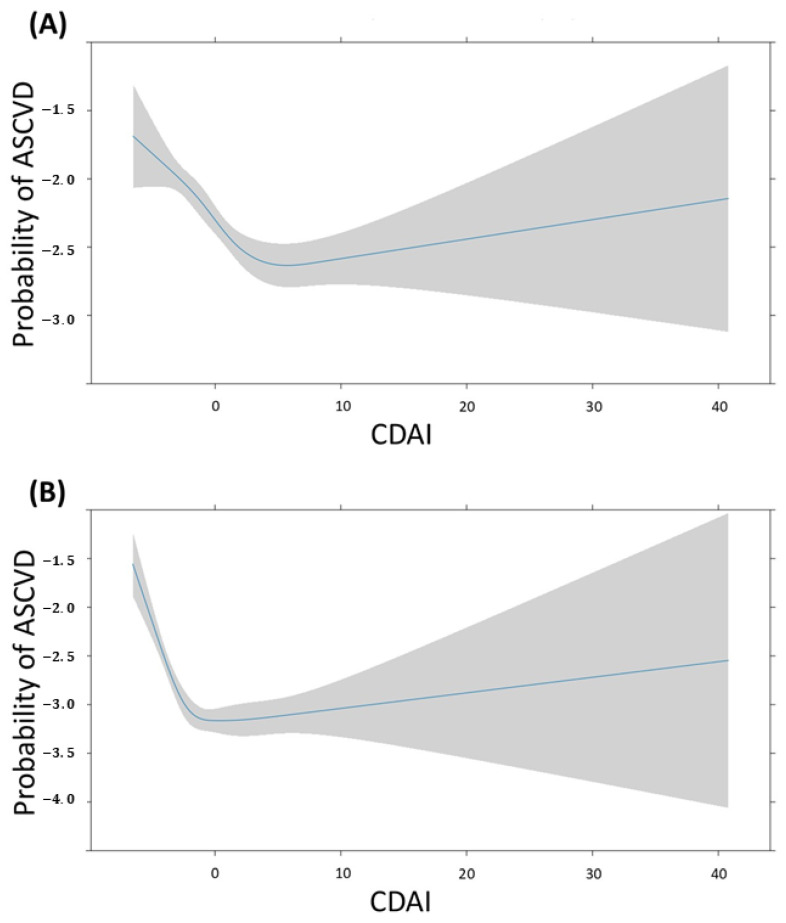
Restricted cubic spline fit of CDAI on ASCVD according to sex. The probability of ASCVD is expressed as log odds. (**A**) Male, (**B**) Female.

Figure 7 depicts the association between CDAI and the probability of ASCVD in males and females. The change in this probability is a non-linear pattern, as the CDAI increases in both groups. For males, the probability is high when the CDAI is <5, it gradually decreases when the CDAI is around 5, and subsequently increases as the CDAI continues to rise. For females, the probability is high when the CDAI is <0, it gradually decreases when the CDAI is 0, and subsequently increases as the CDAI continues to rise. The confidence interval for probability of ASCVD gradually widens after the CDAI reaches 0 in both groups.

## 4. Discussion

In this study, scatter plot and box plot analyses of the CDAI revealed higher levels in people without ASCVD than in those with ASCVD. However, when examining the CDAI as a continuous variable with covariate adjustments, no significant association was found with ASCVD. In the restricted cubic spline, the probability of ASCVD decreases steeply from the minimum value to around 0, and then gradually increases. This non-linear pattern appears in both males and females, although the turning point values may differ. Notably, a significant association emerged when the CDAI was categorized into quartiles. The third quartile exhibited a lower prevalence risk of ASCVD compared with the first one. Interestingly, this association demonstrated heterogeneity based on sex, indicating that the CDAI increased the risk of ASCVD in women but reduced it in men. These findings underscore a nuanced relationship between antioxidants (as indicated by CDAI levels) and cardiovascular health. This relationship appears to be intricate and contingent on varying levels of antioxidants and an individual’s sex.

Oxidative stress has been associated with the pathogenesis of atherosclerosis and coronary artery disease [26]. Antioxidants reduce the prevalence of ASCVD by capturing and deactivating free radicals, which can result in tissue damage. Antioxidants can slow down or prevent plaque formation by inhibiting low-density lipoprotein oxidation, regulating platelet activity, reducing thrombogenicity, and regulating vascular reactivity [27,28,29,30,31,32,33]. Several observational studies have also found that antioxidant intake is associated with a lower risk of major cardiovascular events [34,35,36]. However, recent studies have produced inconsistent results. Mirmiran et al. followed up 5000 people over the age of 30 in Iran for 5.3 years and found that, among vitamins A, C, and E and zinc, only vitamin E was associated with a lower CVD risk [37]. By contrast, Yin et al. reported that vitamins A, C, and E, beta-carotene, and zinc were all associated with a lower risk of CVD in a cross-sectional study of 39,757 adults (≥20 years of age) in the National Health and Nutrition Examination Survey from 2005 to 2018 [38]. Liu et al. conducted a study using data from the National Health and Nutrition Examination Survey from 2013 to 2018. Their study, which was conducted using a similar method to ours, found that the CDAI, which includes zinc, selenium, carotenoids, vitamin A, vitamin C, and vitamin E, was associated with a lower risk of ASCVD in postmenopausal women [8]. In a study conducted on men aged ≥ 75 years in Australia, there was no significant association between antioxidants (vitamins A, C, and E and zinc) and major adverse cardiovascular events [39]. Ours and other studies have shown results that varied with the country, age, sex, and specific population studied. Conflicting findings have been reported, with some studies suggesting a protective effect of antioxidants against cardiovascular disease and others finding no statistically significant association.

There is a strong correlation between oxidative stress and metabolic disorders. Obesity and insulin resistance, which are key components of the metabolic syndrome, are particularly closely linked to increased oxidative stress [40]. Specifically, visceral fat accumulation leads to an increase in the levels of reactive oxygen species, inducing oxidative stress [41]. Inflammatory cytokines secreted from adipose tissue can also promote oxidative stress [42]. Insulin resistance is a condition in which cells become less responsive to insulin, impairing blood glucose control [43]. During this process, mitochondrial energy metabolism is disrupted, leading to increased reactive oxygen species production and oxidative stress [44], which can in turn interfere with insulin signaling, exacerbating insulin resistance in a vicious cycle [45]. Consequently, the increased oxidative stress associated with the metabolic syndrome can increase the risk of developing cardiovascular disease, diabetes, and other complications.

The findings of our study differed in several aspects from those of other studies. First, in continuous data, there was no significant association between increasing CDAI and reduced ASCVD. However, regarding ordinal data, only the third quartile of CDAI showed a significant association with reduced ASCVD. By contrast, the fourth quartile of CDAI, which represents those who consumed more antioxidants, did not show a significant association with reduced ASCVD. In our study, antioxidant intake was assessed based on food consumption. Additionally, in the restricted cubic spline model, the probability of ASCVD changed non-linearly as the CDAI increased. When the CDAI was either very low or very high, the probability of ASCVD increased. In particular, the probability of ASCVD was high with very low CDAI. However, this association is not a straightforward one, and further investigation is needed to understand how the risk of ASCVD changes across intermediate CDAI ranges.

Another notable finding from our study is that the effect of CDAI on ASCVD in men differs from that in women. For men, there was an inverse correlation between the CDAI and ASCVD; whereas, for women, there was a direct correlation between the two. A prospective study of 1605 men found that vitamin deficiency was a risk factor for coronary heart disease [46]. However, a large, long-term trial of vitamin E and vitamin C supplementation in male physicians did not find that these supplements reduced the risk of major adverse cardiovascular events [47]. The effects of individual antioxidants on CVD in women were also heterogeneous in previous studies. A study of 85,118 female nurses in the United States, which was published in 2003, found that vitamin C supplementation was associated with a decreased risk of CVD during a 16-year follow-up period [48]. Another large prospective cohort study conducted in Japan found an inverse correlation between vitamin C intake and CVD incidence [49]. A prospective study of 34,486 postmenopausal women provides evidence of an inverse correlation between dietary vitamin E intake and coronary heart disease incidence; however, no significant correlation was observed for vitamin A or C [50]. Nevertheless, other studies have shown conflicting results. High vitamin C intake has been associated with an increased risk of cardiovascular disease mortality in diabetic postmenopausal women [51]. Another prospective study demonstrated that a higher total zinc intake was associated with an increased risk of cardiovascular disease incidence in women aged > 50 years [52]. These complex and sometimes conflicting results suggest that multiple factors, including sex, dietary patterns, and individual health status, may influence how antioxidants impact cardiovascular health.

The observed sex-specific association between antioxidant intake (as measured by the CDAI) and ASCVD prompts a need to explore the potential mechanisms that underlie these differences. While the precise mechanisms are not yet fully elucidated, several factors may contribute to this intriguing phenomenon. One crucial factor to consider is the influence of sex hormones. Estrogens, which are predominantly present in women, have been shown to have a multifaceted impact on cardiovascular health [53]. They can affect lipid profiles, endothelial cell function, vascular reactivity, and homeostatic factors, and even act as antioxidants themselves [54]. This means that in women, the effect of the CDAI on the incidence of ASCVD may be more nuanced due to the interplay of these hormonal factors. Furthermore, baseline levels of oxidative stress in men may differ from those in women, as it is documented that the levels are often higher in men than in women [55,56]. As a result, the effect of antioxidant intake could hold more significance in the context of ASCVD risk reduction in men.

Our study possesses several strengths that enable it to contribute significantly to the existing knowledge base. Notably, it sheds light on sex-specific differences in how antioxidants (assessed through the CDAI) impact ASCVD, thereby providing valuable insights into sex-specific effects. Additionally, the substantial sample size (19,818 individuals) bolsters the statistical robustness and enhances the generalizability of our findings. Furthermore, our dataset encompassed crucial information on inflammation-related markers such as white blood cells and platelets. This inclusion offers a deeper understanding of the intricate relationship between dietary antioxidant intake, inflammation, and oxidative markers. These additions can significantly augment our capacity to explore the complex interplay among these factors. Nevertheless, our study has several limitations that should be acknowledged. First, its cross-sectional design restricts our ability to establish causality. While we identified an association between CDAI and the prevalence of ASCVD, establishing direct causality remains challenging. Second, our assessment of antioxidant intake relied on dietary information (primarily through dietary recall or food frequency questionnaires), which may be susceptible to recall bias and underreporting of specific foods, and variations can occur if measurements are performed multiple times for the same subject. The accuracy of estimating antioxidant intake from such data could potentially impact our results. Third, despite employing statistical adjustments, the presence of unmeasured or residual confounding factors might still influence the observed association between CDAI and the prevalence of ASCVD. Fourth, although our study highlights sex-specific differences, it does not deeply explore the underlying mechanisms driving these distinctions. Further research is necessary to comprehensively grasp the reasons behind these observed sex-based variations. Fifth, atherosclerosis can block arteries and compromise blood flow, and it can equally induce thrombosis. It is a common cause of heart attack, stroke, and peripheral artery disease (PAD). Unfortunately, due to the absence of PAD data, our study did not include PAD. Analyzing ASCVD, including PAD, would have confirmed the clarity of the CDAI effect. Future studies with analyses that include PAD are warranted.

## 5. Conclusions

We investigated the complex relationship between antioxidant intake (as measured using the CDAI) and the prevalence of ASCVD in this comprehensive study. Our study revealed intriguing patterns that vary depending on antioxidant levels and sex, demonstrating the complexity of these relationships. These findings highlight the importance of a personalized approach to cardiovascular risk assessment and intervention, as such an approach would focus on an individual’s antioxidant profile and sex-specific risk factors. Additional research should be conducted to elucidate the underlying mechanisms and improve our understanding of the complex interplay between antioxidants and cardiovascular health.

## Figures and Tables

**Table 1:** Baseline Characteristics of the Study Population from National Health and Nutrition Examination Survey 2016–2021.

Variables		None ASCVD (*n* = 18,436)	ASCVD (*n* = 1382)	ASD
Survey year		2018 (2017–2020)	2019 (2017–2020)	0.03
Age, years	40–49	4904 (26.6)	53 (3.8)	1.00
	50–59	4938 (26.8)	179 (13)	
	60–69	4665 (25.3)	403 (29.2)	
	70–79	3027 (16.4)	557 (40.3)	
	80<=	902 (4.9)	190 (13.7)	
Sex	Male	7626 (41.4)	788 (57.0)	0.32
	Female	10,810 (58.6)	594 (43.0)	
Weight, kg		61.9 (54.9–70.4)	63.1 (56.1–71.5)	0.09
Waist circumference, cm		84.1 (77.3–90.4)	87.9 (81.8–94.2)	0.42
Body mass index, kg/m^2^		23.9 (21.8–26.1)	24.6 (22.5–26.6)	0.19
Weight change over 1 year	No change	12,622 (68.5)	933 (67.5)	0.05
	Decrease	2194 (11.9)	241 (17.4)	
	Increase	3620 (19.6)	208 (15.1)	
Household income quartiles	Low	3889 (21.1)	536 (38.8)	0.49
	Lower-middle	4552 (24.7)	381 (27.6)	
	Upper-middle	4719 (25.6)	266 (19.2)	
	Higher	5276 (28.6)	199 (14.4)	
Educational attainment	Below elementary school graduate	4494 (24.4)	602 (43.6)	0.54
	Junior high school graduate	2328 (12.6)	249 (18)	
	High school graduate	5839 (31.7)	330 (23.9)	
	College graduate or higher	5775 (31.3)	201 (14.5)	
Marital status	Not married	696 (3.8)	38 (2.7)	0.18
	Married, living together	14,451 (78.4)	973 (70.4)	
	Married, living apart	168 (0.9)	17 (1.2)	
	Widowed	1984 (10.8)	269 (19.5)	
	Divorced	1137 (6.2)	85 (6.2)	
Type of health insurance	NHI, local	5800 (31.5)	439 (31.8)	0.08
	NHI, employment	11,861 (64.3)	807 (58.4)	
	Medical aid	727 (3.9)	131 (9.5)	
	Not enrolled, Do not know, no answer	48 (0.3)	5 (0.4)	

ASCVD, atherosclerotic cardiovascular disease; ASD, absolute standardized difference; NHI, National Health Insurance.

**Table 2:** Odds Ratio of CDAI on the Prevalence of Atherosclerotic Cardiovascular Disease.

Item	Adjusting Variables	Reference	CDAI Quartile	OR (95%CI)	*p*-Value
CDAI	Unadjusted			0.96 (0.94–0.99)	0.002
	Fully Adjusted			1.0 (0.98–1.02)	0.954
Quartile of CDAI	Unadjusted	CDAI 1st quartile	CDAI 2nd quartile	0.73 (0.61–0.87)	<0.001
			CDAI 3rd quartile	0.55 (0.46–0.66)	<0.001
			CDAI 4th quartile	0.61 (0.51–0.74)	<0.001
	Fully Adjusted	CDAI 1st quartile	CDAI 2nd quartile	0.94 (0.77–1.15)	0.54
			CDAI 3rd quartile	0.77 (0.62–0.95)	0.014
			CDAI 4th quartile	0.95 (0.76–1.2)	0.674

CDAI, composite dietary antioxidant index; OR, odds ratio; CI, confidence interval.

**Table 3:** Odds Ratio of CDAI According to the Prevalence of Atherosclerotic Cardiovascular Disease in Males and Females.

Item	Adjusting Variables	Group	Reference	CDAI Quartile	OR (95%CI)	*p*-Value
CDAI	Unadjusted	Male			0.94 (0.91–0.97)	<0.001
		Female			0.96 (0.92–1)	0.042
	Fully Adjusted	Male			0.99 (0.96–1.01)	0.33
		Female			1.02 (0.99–1.06)	0.153
Quartile of CDAI	Unadjusted	Male	CDAI 1st quartile	CDAI 2nd quartile	0.71 (0.56–0.92)	0.009
				CDAI 3rd quartile	0.48 (0.38–0.62)	<0.001
				CDAI 4th quartile	0.43 (0.34–0.56)	<0.001
		Female	CDAI 1st quartile	CDAI 2nd quartile	0.62 (0.48–0.8)	<0.001
				CDAI 3rd quartile	0.48 (0.36–0.63)	<0.001
				CDAI 4th quartile	0.72 (0.53–0.97)	0.028
	Fully Adjusted	Male	CDAI 1st quartile	CDAI 2nd quartile	0.91 (0.69–1.2)	0.507
				CDAI 3rd quartile	0.71 (0.53–0.94)	0.018
				CDAI 4th quartile	0.75 (0.56–1)	0.053
		Female	CDAI 1st quartile	CDAI 2nd quartile	0.92 (0.7–1.21)	0.556
				CDAI 3rd quartile	0.81 (0.59–1.1)	0.174
				CDAI 4th quartile	1.4 (1.01–1.95)	0.042

CDAI, composite dietary antioxidant index; OR, odds ratio; CI, confidence interval.

**Table 4:** Odds Ratio of CDAI in Relation to the Prevalence of coronary artery diseases and stroke.

	Item	Adjusting Variables	Reference	CDAI Quartile	OR (95%CI)	*p*-Value
Coronary artery diseases	CDAI	Unadjusted			0.98 (0.96–1.00)	0.114
		Fully Adjusted			0.98 (0.96–1.01)	0.166
	Quartile of CDAI	Unadjusted	CDAI 1st quartile	CDAI 2nd quartile	0.74 (0.59–0.94)	0.012
				CDAI 3rd quartile	0.90 (0.72–1.12)	0.367
				CDAI 4th quartile	0.69 (0.55–0.86)	<0.001
		Fully Adjusted	CDAI 1st quartile	CDAI 2nd quartile	0.74(0.58–0.94)	0.012
				CDAI 3rd quartile	0.90 (0.72–1.13)	0.37
				CDAI 4th quartile	0.69 (0.53–0.88)	0.003
Storke	CDAI	Unadjusted			0.98 (0.95–1.01)	0.191
		Fully Adjusted			0.99 (0.96–1.01)	0.282
	Quartile of CDAI	Unadjusted	CDAI 1st quartile	CDAI 2nd quartile	0.82 (0.64–1.05)	0.121
				CDAI 3rd quartile	0.76 (0.58–0.99)	0.048
				CDAI 4th quartile	0.81 (0.62–1.07)	0.141
		Fully Adjusted	CDAI 1st quartile	CDAI 2nd quartile	0.83 (0.64–1.07)	0.145
				CDAI 3rd quartile	0.77 (0.58–1.01)	0.061
				CDAI 4th quartile	0.83 (0.63–1.09)	0.184

CDAI, composite dietary antioxidant index; OR, odds ratio; CI, confidence interval.

## Data Availability

The data presented in this study are openly available in KNHANES at [https://knhanes.kdca.go.kr/knhanes/sub04/sub04_04_01.do] (accessed on 26 August 2024) [14].

## Appendix A

**Table A1:** Overview of Drug Use and Lifestyle Behavior-Related Factors from the National Health and Nutrition Examination Survey 2016–2021.

Variables		None ASCVD (*n* = 18,436)	ASCVD (*n* = 1382)	ASD
Diagnosis of hypertension by a licensed physician		5717 (31)	883 (63.9)	0.68
Diagnosis of dyslipidemia by a licensed physician		4710 (25.5)	633 (45.8)	0.41
Ongoing dyslipidemia medication	Take every day	3237 (17.6)	521 (37.7)	0.43
	Take c20 days a month	86 (0.5)	6 (0.4)	
	Take ≥15 days a month	47 (0.3)	4 (0.3)	
	Take ≤15 days a month	37 (0.2)	1 (0.1)	
	Do not take	1303 (7.1)	101 (7.3)	
	Not applicable (not diagnosed by licensed physician)	13,726 (74.5)	749 (54.2)	
Diagnosis of diabetes by a licensed physician		2265 (12.3)	405 (29.3)	0.37
Drinking frequency over the past year	Did not drink at all in the past 1 year	2482 (13.5)	261 (18.9)	0.15
	Less than once a month	3651 (19.8)	365 (26.4)	
	Approximately once a month	3453 (18.7)	193 (14)	
	2–4 times a month	1634 (8.9)	94 (6.8)	
	About 2–3 times a week	3431 (18.6)	197 (14.3)	
	≥4 times a week	2506 (13.6)	156 (11.3)	
	Not applicable	1279 (6.9)	116 (8.4)	
Amount of alcohol consumed in one sitting	1–2 glasses	6133 (33.3)	626 (45.3)	0.21
	3–4 glasses	5237 (28.4)	328 (23.7)	
	5–6 glasses	2688 (14.6)	182 (13.2)	
	7–9 glasses	1598 (8.7)	83 (6)	
	≥10 glasses	1708 (9.3)	108 (7.8)	
	Not applicable	1072 (5.8)	55 (4)	
Taking at least one drink per month in the past year		8850 (48)	563 (40.7)	0.15
Average daily cigarette smoking	No smoking	15,700 (85.2)	1165 (84.3)	0.03
	≤20 cigarettes	2550 (13.8)	199 (14.4)	
	>20 cigarettes	186 (1)	18 (1.3)	
Typical daily sitting time, hours	≤4 h	1778 (9.6)	74 (5.4)	0.26
	≤8 h	6726 (36.5)	434 (31.4)	
	≤12 h	6506 (35.3)	493 (35.7)	
	>12 h	3426 (18.6)	381 (27.6)	
Practicing aerobic physical activity		7344 (39.8)	443 (32.1)	0.17
Number of days of vigorous physical activity (work)	None	18,231 (98.9)	1367 (98.9)	<0.01
	1 day	34 (0.2)	1 (0.1)	
	2 days	39 (0.2)	4 (0.3)	
	3 days	30 (0.2)	2 (0.1)	
	4 days	17 (0.1)	3 (0.2)	
	5 days	33 (0.2)	2 (0.1)	
	6 days	26 (0.1)	1 (0.1)	
	7 days	26 (0.1)	2 (0.1)	
Number of days of moderate-intensity physical activity (work)	None	17,418 (94.5)	1307 (94.6)	0.03
	1 day	151 (0.8)	13 (0.9)	
	2 days	187 (1)	16 (1.2)	
	3 days	205 (1.1)	19 (1.4)	
	4 days	101 (0.5)	11 (0.8)	
	5 days	188 (1)	6 (0.4)	
	6 days	72 (0.4)	1 (0.1)	
	7 days	114 (0.6)	9 (0.7)	
Physical activity status: Moving around	None	9005 (48.8)	765 (55.4)	0.12
	1 day	721 (3.9)	50 (3.6)	
	2 days	1096 (5.9)	70 (5.1)	
	3 days	1690 (9.2)	134 (9.7)	
	4 days	869 (4.7)	60 (4.3)	
	5 days	2020 (11)	94 (6.8)	
	6 days	599 (3.2)	33 (2.4)	
	7 days	2436 (13.2)	176 (12.7)	
Number of days of vigorous physical activity (leisure)	None	17,059 (92.5)	1347 (97.5)	0.25
	1 day	239 (1.3)	2 (0.1)	
	2 days	240 (1.3)	12 (0.9)	
	3 days	356 (1.9)	6 (0.4)	
	4 days	187 (1)	5 (0.4)	
	5 days	219 (1.2)	4 (0.3)	
	6 days	66 (0.4)	3 (0.2)	
	7 days	70 (0.4)	3 (0.2)	
Number of days of moderate-intensity physical activity (leisure)	None	14,193 (77)	1165 (84.3)	0.13
	1 day	503 (2.7)	15 (1.1)	
	2 days	742 (4)	44 (3.2)	
	3 days	1131 (6.1)	40 (2.9)	
	4 days	572 (3.1)	32 (2.3)	
	5 days	730 (4)	47 (3.4)	
	6 days	194 (1.1)	16 (1.2)	
	7 days	371 (2)	23 (1.7)	
Number of strength-training days per week	None	14,186 (76.9)	1100 (79.6)	0.02
	1 day	504 (2.7)	29 (2.1)	
	2 days	800 (4.3)	45 (3.3)	
	3 days	982 (5.3)	44 (3.2)	
	4 days	399 (2.2)	27 (2)	
	≥5 days	1565 (8.5)	137 (9.9)	
Glycated hemoglobin level, mg/dL		5.7 (5.4–6)	6 (5.675–6.5)	0.38
Total Cholesterol level, mg/dL		194 (168–219)	160 (136–190)	0.73
High-density lipoprotein level, mg/dL		50 (42–58.5)	45.9 (39–54)	0.32
Triglyceride level, mg/dL		112 (78–164)	116.5 (81–163)	0.02
White blood cell count, 1000/uL		5.84 (4.89–6.97)	6.125 (5.2–7.22)	0.19
Platelet count, 1000/uL		249 (212–290)	234 (196–277.25)	0.19

**Table A2:** Adjusted Odds Ratios and 95% Confidence Intervals of Covariates for Continuous and Ordinal CDAI data.

	Continuous CDAI		Ordinal CDAI	
Variables	Adjusted OR (95%CI)	*p*-Value	Adjusted OR (95%CI)	*p*-Value
Survey year	0.97 (0.93–1.01)	0.136	0.97 (0.93–1.01)	0.138
Sex	0.46 (0.36–0.58)	<0.001	0.46 (0.36–0.58)	<0.001
Age, years	1.58 (1.44–1.73)	<0.001	1.58 (1.44–1.73)	<0.001
Household income quartiles	0.96 (0.89–1.04)	0.324	0.97 (0.89–1.04)	0.389
Educational attainment	0.94 (0.86–1.01)	0.099	0.94 (0.87–1.02)	0.12
Marital status	1.01 (0.93–1.09)	0.84	1.01 (0.93–1.09)	0.871
Type of health insurance	1.01 (1–1.01)	0.144	1.01 (1–1.01)	0.165
Diagnosis of hypertension by a licensed physician	1.77 (1.5–2.1)	<0.001	1.77 (1.5–2.1)	<0.001
Diagnosis of dyslipidemia by a licensed physician	1.76 (1.11–2.78)	0.016	1.78 (1.12–2.81)	0.014
Ongoing dyslipidemia treatment	1.05 (0.97–1.13)	0.2	1.05 (0.98–1.13)	0.194
Diagnosis of diabetes by a licensed physician	0.93 (0.75–1.16)	0.529	0.93 (0.75–1.16)	0.522
Weight change over 1 year	1.09 (0.99–1.21)	0.071	1.09 (0.99–1.2)	0.077
Drinking frequency over 1 year	0.99 (0.9–1.09)	0.864	0.99 (0.9–1.09)	0.854
Amount of alcohol consumed in one sitting	0.91 (0.84–1)	0.038	0.91 (0.84–0.99)	0.037
Drinking at least 1 drink per month in the past year	1.01 (0.72–1.42)	0.939	1.02 (0.73–1.43)	0.906
Average daily cigarette smoking	0.95 (0.78–1.15)	0.615	0.95 (0.78–1.15)	0.588
Number of days of vigorous physical activity (work)	1.02 (0.88–1.17)	0.83	1.02 (0.88–1.18)	0.809
Number of days of moderate-intensity physical activity (work)	1.03 (0.95–1.11)	0.474	1.03 (0.95–1.11)	0.491
Physical activity status: Moving around	0.98 (0.94–1.01)	0.188	0.98 (0.94–1.01)	0.189
Number of days of vigorous physical activity (leisure)	0.89 (0.79–1.01)	0.06	0.89 (0.79–1.01)	0.061
Number of days of moderate-intensity physical activity (leisure)	1.02 (0.97–1.07)	0.454	1.02 (0.97–1.07)	0.437
Typical daily sitting time, hours	1.17 (1.09–1.27)	<0.001	1.17 (1.08–1.26)	<0.001
Number of strength-training days per week	0.98 (0.93–1.02)	0.347	0.98 (0.94–1.03)	0.386
Practicing aerobic physical activity	1.02 (0.81–1.27)	0.892	1.01 (0.81–1.26)	0.918
Weight, kg	0.99 (0.98–1.01)	0.484	1 (0.98–1.01)	0.57
Waist circumference, cm	1.01 (1–1.03)	0.138	1.01 (1–1.03)	0.148
Body mass index, kg/m^2^	1.01 (0.96–1.07)	0.668	1.01 (0.96–1.07)	0.716
Glycated hemoglobin level, mg/dL	1.16 (1.07–1.25)	<0.001	1.16 (1.07–1.25)	<0.001
Total Cholesterol level, mg/dL	0.98 (0.98–0.99)	<0.001	0.98 (0.98–0.99)	<0.001
High-density lipoprotein level, mg/dL	1.01 (1.01–1.02)	<0.001	1.01 (1.01–1.02)	<0.001
Triglyceride level, mg/dL	1 (1–1)	<0.001	1 (1–1)	<0.001
White blood cell count, 1000/uL	1.01 (0.97–1.05)	0.533	1.01 (0.97–1.05)	0.574
Platelet count, 1000/uL	1 (1–1)	0.829	1 (1–1)	0.85

CDAI, composite dietary antioxidant index; CI confidence interval; OR, odds ratio.

**Table A3:** Description of Covariates.

Name of Variables	Variable Description	Content
year	Year of survey	Year of survey
age	Age	1~79. 1~79 years old 80. 80 years of age or older
sex	Sex	1. male 2. female
Weight	Weight	Kg
Waist circumference	Waist circumference	Cm
Body mass index	Body mass index	kg/m^2^
Weight change over 1 year	Whether weight changes over a year	1. No change 2. Weight loss 3. Weight gain 8. Not applicable (<6 years old) 9. Do not know, no response
Household income quartiles	Income quartile (household) Refer to the base amount for classifying the fourth quartile	1. low 2. low-to-medium 3. medium-to-high 4. high
Educational attainment	Training Level reclassification code Graduation is classified as the current academic background, and completion, retirement, leave of absence/leave of absence is classified as the previous academic background	1. Elementary school or less 2. Middle school graduate 3. High school graduate 4. College graduate or higher
Marital status	Marital status	1. Married, living together 2. Married, separated 3. Widowed 4. Divorced 8. Refused to answer 9. Do not know 88. Not applicable 99. No response
Type of health insurance	Type of health insurance	10. National Health Insurance (Local) 20. National Health Insurance (Workplace) 30. Medical Assistance 40. Not enrolled, do not know, no response
Drinking frequency over the past year	Drinking frequency for a year	1. Did not drink at all in the past 1 year 2. Less than once a month 3. About once a month 4. 2–4 times a month 5. About 2–3 times a week 6. ≥4 times a week 8. Not applicable 9. Do not know, no answer
Amount of alcohol consumed in one sitting	the amount of alcohol consumed at a time	1. 1–2 glasses 2. 3–4 glasses 3. 5–6 glasses 4. 7–9 glasses 5. ≥10 glasses 8. Not applicable 9. Do not know, no answer
Drinking per month in the past year	Drinking frequency for a year	1. Did not drink alcohol at all in the past 1 year 2. Less than once a month 3. Once a month 4. 2–4 times a month 5. 2–3 times a week 6. ≥4 times a week 8. Not applicable 9. Do not know, no answer
Smoking status	Average daily cigarette smoking	□□ cigarettes 88. Not applicable (adolescents or children or never smoked) 9. Do not know, no answer
Typical daily sitting time	Typical daily sitting time	hours
Practicing aerobic physical activity	Aerobic Physical Activity Practice Rate	0. Did not engage in the recommended amount of physical activity, which is at least 2.5 h of moderate-intensity physical activity or 1.5 h of vigorous-intensity physical activity per week, or an equivalent combination of moderate- and vigorous-intensity activity (1 min of vigorous-intensity activity is equal to 2 min of moderate-intensity activity) 1. Engaged in the recommended amount of physical activity, which is at least 2.5 h of moderate-intensity physical activity or 1.5 h of vigorous-intensity physical activity per week, or an equivalent combination of moderate- and vigorous-intensity activity (1 min of vigorous-intensity activity is equal to 2 min of moderate-intensity activity)
Number of days of vigorous physical activity (work)	High-intensity physical activity days: days	□□ days 88. Not applicable (adolescents, children or no high-intensity physical activity) 9. Do not know, no answer
Number of days of moderate-intensity physical activity (work)	Medium-intensity number of physical activity days: days	□□ days 88. Not applicable (adolescents, children or no moderate-intensity physical activity) 9. Do not know, no answer
Physical activity status: Moving around	Physical activity: Moving to a location	1. Yes 2. No 8. Not applicable 9. Do not know, no answer
Number of days of vigorous physical activity (leisure)	High-intensity physical activity days: days	□□ days 88. Not applicable (adolescents, children or no high-intensity physical activity.) 9. Do not know, no answer
Number of days of moderate-intensity physical activity (leisure)	Medium-intensity number of physical activity days: days	□□ days 88. Not applicable (adolescents, children or no moderate-intensive physical activity.) 9. Do not know, no answer
Number of strength-training days per week	Number of strength-training days per week	1. Not at all 2. 1 day 3. 2 days 4. 3 days 5. 4 days 6. ≥5 days 8. Not applicable 9. Do not know, no answer
Diagnosis of hypertension by a licensed physician	Doctor’s diagnosis of hypertension	0. None 1. Yes 8. Not applicable (adolescents, children) 9. Do not know, no response
Diagnosis of diabetes by a licensed physician	Diagnosis of diabetes by a licensed physician	0. None 1. Yes 8. Not applicable (adolescents, children) 9. Do not know, no response
Glycated hemoglobin level	Glycated hemoglobin level	mg/dL
Total cholesterol level	total cholesterol level	mg/dL
Ongoing dyslipidemia treatment	Taking dyslipidemia medication	Take every day Take ≥20 days a month Take ≥15 days a month Take ≤15 days a month Do not take Not applicable (not diagnosed by a licensed physician) Do not know, no response
High-density lipoprotein level	High-density lipoprotein level	mg/dL
Low-density lipoprotein level	Low-density lipoprotein level	mg/dL
Triglyceride level	Triglyceride level	mg/dL
White blood cell count	White blood cell count	1000/uL
Platelet count	Platelet count	1000/uL
